# 3-Dimensional Examination of the Adult Mouse Subventricular Zone Reveals Lineage-Specific Microdomains

**DOI:** 10.1371/journal.pone.0049087

**Published:** 2012-11-15

**Authors:** Kasum Azim, Roberto Fiorelli, Stefan Zweifel, Anahi Hurtado-Chong, Kazuaki Yoshikawa, Lutz Slomianka, Olivier Raineteau

**Affiliations:** 1 Brain Research Institute, University of Zürich/ETHZ, Zürich, Switzerland; 2 Institute of Anatomy, University of Zürich, Zürich, Switzerland; 3 Institute for Protein Research Osaka University, Osaka, Japan; Seattle Children’s Research Institute, United States of America

## Abstract

Recent studies suggest that the subventricular zone (SVZ) of the lateral ventricle is populated by heterogeneous populations of stem and progenitor cells that, depending on their exact location, are biased to acquire specific neuronal fates. This newly described heterogeneity of SVZ stem and progenitor cells underlines the necessity to develop methods for the accurate quantification of SVZ stem and progenitor subpopulations. In this study, we provide 3-dimensional topographical maps of slow cycling “stem” cells and progenitors based on their unique cell cycle properties. These maps revealed that both cell populations are present throughout the lateral ventricle wall as well as in discrete regions of the dorsal wall. Immunodetection of transcription factors expressed in defined progenitor populations further reveals that divergent lineages have clear regional enrichments in the rostro-caudal as well as in the dorso-ventral span of the lateral ventricle. Thus, progenitors expressing Tbr2 and Dlx2 were confined to dorsal and dorso-lateral regions of the lateral ventricle, respectively, while Mash1+ progenitors were more homogeneously distributed. All cell populations were enriched in the rostral-most region of the lateral ventricle. This diversity and uneven distribution greatly impede the accurate quantification of SVZ progenitor populations. This is illustrated by measuring the coefficient of error of estimates obtained by using increasing section sampling interval. Based on our empirical data, we provide such estimates for all progenitor populations investigated in this study. These can be used in future studies as guidelines to judge if the precision obtained with a sampling scheme is sufficient to detect statistically significant differences between experimental groups if a biological effect is present. Altogether, our study underlines the need to consider the SVZ of the lateral ventricle as a complex 3D structure and define methods to accurately assess neural stem cells or progenitor diversity and population sizes in physiological or experimental paradigms.

## Introduction

The subventricular zone (SVZ, also referred to as the subependymal zone, SEZ) of the lateral ventricle (LV) of the forebrain is a prominent neurogenic niche in the adult CNS and is of prime importance in studying cell differentiation and specification programmes. Subependymal stem cells (type-B cells) express GFAP and locally give rise to fast proliferating progenitors (type-C cells) that eventually differentiate into committed neuroblasts (type-A cells) [1]. These neuroblasts migrate as protracted chains termed the rostral migratory stream (RMS) until they reach the olfactory bulb to differentiate into phenotypically and functionally distinct populations of neurons in the granule and glomerular layers [2].

Accumulating evidence underlines major differences in the time and place of origin of distinct olfactory neuronal subtypes (reviewed in [2], [3]). Original work that fuelled these observations was the demonstration, by retroviral labelling, of progenitors in the LV or the RMS resulting in the generation of granular or periglomerular cells respectively [4]–[6]. Subsequent studies showed that distinct olfactory neuronal subtypes originate from defined regions of the LV, both in the early postnatal [7], [8] and adult forebrain [8]–[12]. In the adult forebrain, most newly generated olfactory bulb neurons are superficial granule neurons as well as subclasses of GABAergic periglomerular neurons that express either tyrosine hydroxylase or calretinin. These neuronal subclasses originate from the lateral and dorsal walls of the LV respectively.

Consequently, SVZ-neural stem cells (SVZ-NSCs) show characteristics of early restricted progenitors that are biased to acquire specific fates based on their place of origin. These properties appear to be intrinsic to these cells, as heterochronic and/or heterotypic transplantations experiments failed to re-specify them [8], [10], [12], [13]. Defined transcription factors (TFs) are expressed in progenitors of the LV, and are likely to participate in the early specification of SVZ-NSCs towards a specific neuronal fate [9], [11], [12], [14]. The TFs Dlx2 and Mash1 (also known as Ascl1) are expressed in the adult SVZ and are necessary for the generation of most olfactory bulb interneurons originating from the lateral SVZ [14], [15]. Besides its generic role in neurogenesis, evidence supports a role for Dlx2 in neuronal subtype specification in the olfactory bulb [14]. The TF Tbr2 (also referred to as Eomes) is expressed in a subpopulation of progenitors arising from the dorsal SVZ [11] that specify a small population of olfactory bulb glutamatergic neurons. Therefore, progenitor diversity in the SVZ could be identifiable by differential expression of defined TFs.

**Figure 1:**
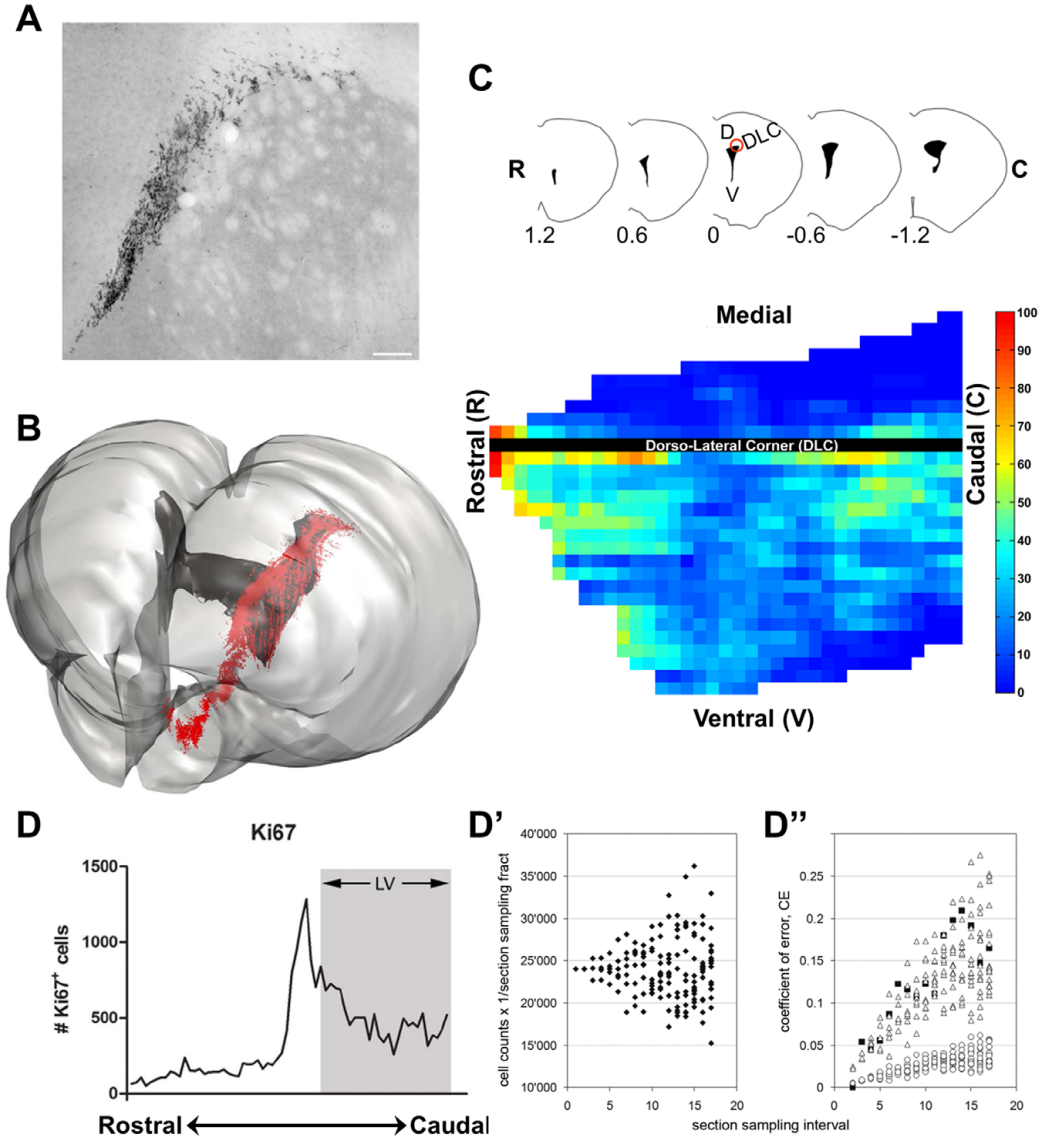
Distribution of proliferating Ki67-positive progenitors in the adult mouse forebrain. (**A**) Photomicrograph shows a representative wide-field image used for generating the 3D model, showing Ki67 immunoreactivity on a section immediately rostral to the LV aperture. Scale bar: 150 µm. (**B**) 3D modelling of the distribution of Ki67+ cells (red cells) along the LV (dark grey shapes) of in the adult mouse forebrain (light grey contours). (**C**) Top cross-sections illustrate the shape of sections and LV at 5 equally spaced rostro-caudal forebrain levels (i.e. 1.2, 0 and −1.2 to bregma). Individual sections were used to generate the flattened heat map below. Each column represents a section from the rostral to the caudal LV, with 100 µm sized squares positioned along the dorsal (rows above the black line representing the dorso-lateral corner (DLC) of the LV) and lateral (rows below the black line). Colours codes show the relative densities of quantified Ki67+ progenitors (higher values: red; lower values: blue). (**D**) Ki67+ cells quantified in individual sections along the anterior-posterior axis of the forebrain (from the olfactory bulb to the caudal-most regions of the LV, as defined in material and methods). (**D’**) Scaled cell counts obtained by subsampling the original counts for section sampling intervals using every 2^nd^ to every 17^th^ sections. (**D”**) Estimates of the CE obtained by using Gundersen-Jensen estimator using a smoothness constant of 0 (open triangles) and 1 (open circles) for section sampling intervals using every 2^nd^ to every 17^th^ sections. Black squares indicate the observed CEs, i.e. the coefficient of variation among the replicates available for each sampling interval. D  =  dorsal; V  =  ventral; DLC  =  dorso-lateral corner; R  =  rostral; C  =  caudal.

In the present study, we focused on the anterior 2/3 of the lateral ventricle, i.e. the most commonly studied neurogenic region of the LV which is phylogenetically maintained in primates [16], [17]. We generated 3D maps of NSCs and progenitors using the proliferative markers Mcm2 and Ki67 (expressed in G1, S, G2 and M cell cycle phases) in combination with S-phase markers (i.e. EdU) distinguishing slow cycling NSCs from fast cycling progenitors [18]. We further assessed the distribution of progenitor populations expressing TFs involved in divergent lineage specification during development, i.e. Mash1, Dlx2 and Tbr2 [19]–[21]. Our results reveal clear regional enrichments of stem and progenitor cells in microdomains of the SVZ. The spatial complexity and dramatically different population sizes of these progenitors require special care to obtain accurate and precise quantitative measures, which are needed to assess the outcomes of experimental intervention in SVZ neurogenesis. Based on these observations, we developed and present here sampling schemes that efficiently address these issues. These sampling schemes will prove to be important to accurately assess changes in NSC/progenitor populations or diversity in other mammalian species as well as in the context of brain repair following a lesion or in various pathologies.

**Figure 2:**
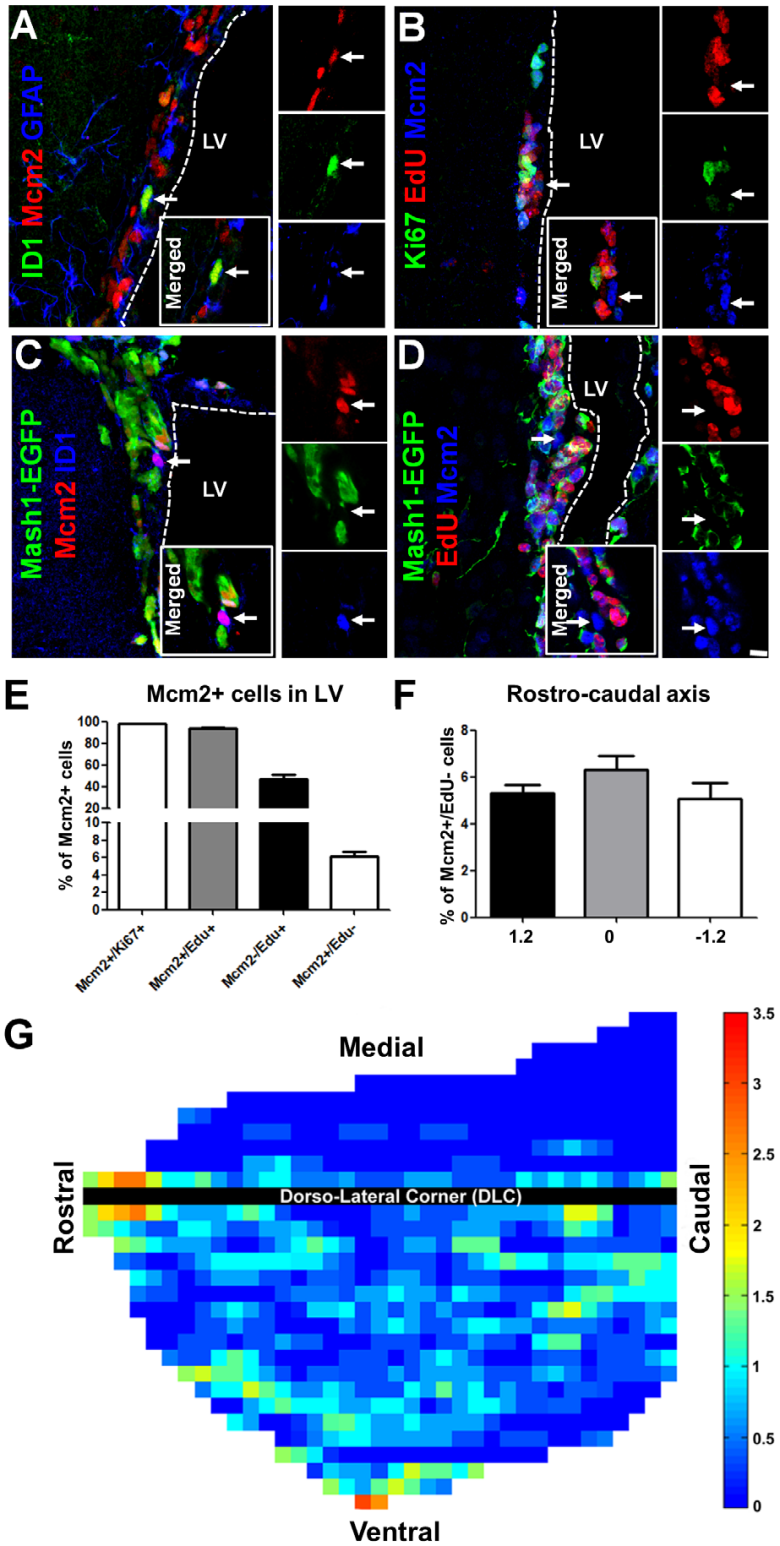
Identification and location of slow-cycling SVZ-NSCs. (**A–D**) Adult wildtype or Mash1 driven EGFP mice were treated with EdU in drinking water for 3 days and rostral sections immunostained for proposed NSCs markers (ID1/GFAP, **A,C**) to illustrate co-expression with Mcm2, but exclusion of S-phase marker EdU (**B**) or Mash1-EGFP (**C,D**). Arrows in full panels indicate examples of typical Mcm2+ NSCs with other marker combinations. Single captions of single channel confocal planes as well as the corresponding merged image are also presented as insets. Dotted lines mark the approximate ventricular space. Full panel flattened confocal z-stacks are of ∼10 µm thickness. Scale bar in **D**: 15 µm in full panels and 20 µm in insets. (**E**) Quantification of Mcm2+ cells expressing other proliferative markers (i.e. Ki67 and EdU). (**F**) Throughout the different rostro-caudal coordinates of the adult SVZ, the percentage of Mcm2+/EdU− NSCs among the entire population of Mcm2+ cycling cells remained constant, suggesting that the distribution of slow cycling cells is identical to the distribution of all cycling cells. (**G**) Flattened heat map showing the distribution of NSCs throughout the dorsal and lateral walls of the LV (as shown in **Fig. 1**). Each column represents a section from the rostral to the caudal LV, with 100 µm sized squares positioned along the dorsal (rows above the black line representing the dorso-lateral corner of the LV) and lateral (rows below the black line). Colours code shows the relative densities of Mcm2+/EdU− NSCs (higher values: red; lower values: blue).

## Experimental Procedures

Unless stated otherwise materials and solutions were obtained from Sigma-Aldrich (Buchs, Switzerland).

### Ethics Statement

All protocols for our experiments were approved by the Ethics Committee of the Veterinary Department of the Canton of Zurich (Approval ID 182/2011). Animal procedures were executed in accordance with Swiss law, with strict consideration given to the care and use of animals.

**Figure 3:**
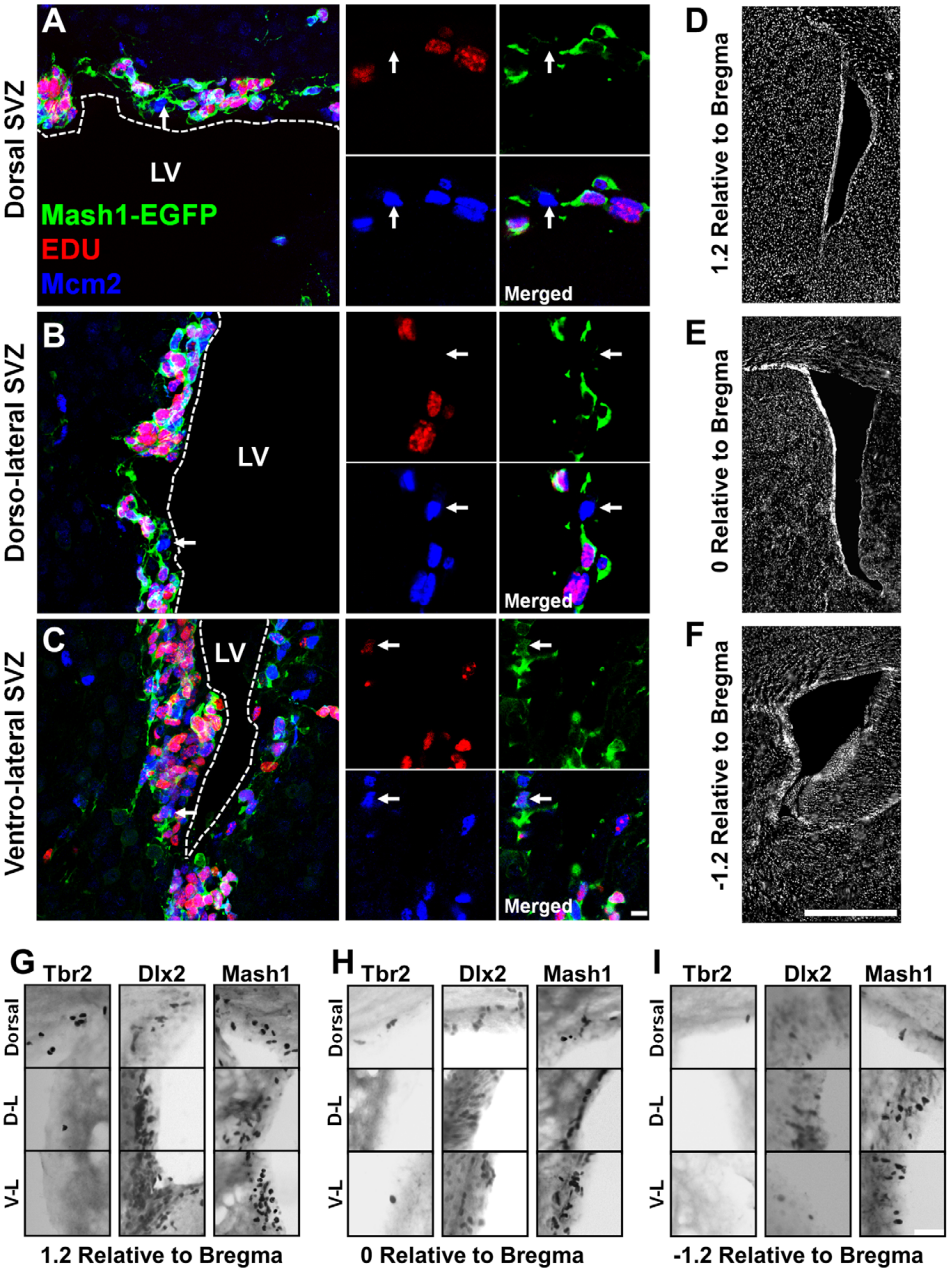
Examples of EGFP−/Mcm2+/EdU− NSCs in rostral SVZ microdomains and lineage restricted progenitors in defined rostro-caudal axis of the LV. Mash1-EGFP mouse were treated with the S-phase marker EdU for 3 days to label actively cycling cells. Forebrain coronal sections were immunostained for Mcm2 (blue) and EdU dye-coupling (red). Arrows indicate typical EGFP−/Mcm2+/EdU- NSC present in each of the individual SVZ microdomains. Exemplified NSCs by arrows are expanded in right captions to show single z-plane channels and the merge of the single z-plane. Dotted lines mark approximate boundaries of ventricular space. Flattened confocal z-stacks are of 14–15 µm thickness whilst captions are single z-planes. Scale bars: 15 µm in full panels and 20****µm in captions. (**D–F**) Right hand panel overviews illustrate merged mosaics of DAPI stained sections at indicated rostro-caudal coordinates (i.e. 1.2, 0 and −1.2). Note: reduction in EGFP intensity compared to **Fig 4** **.** is due to EdU detection protocol containing a bleaching agent. (**G–I**) Photomicrographs of representative DAB staining for Tbr2, Dlx2 and Mash1 in adult SVZ periventricular sections at positions 1.2, 0 and −1.2 relative to the bregma. Captions show zoomed and cropped individual SVZ microdomains. D  =  Dorsal; D-L  =  dorso-lateral; V-L  =  ventro-lateral. Scale bars: 1 mm in **D–F** and 50 µm in **G-**.

### Animal and Tissue Processing

In all experiments, 10 to 12 weeks-old mice of C57/BL6 background were used. These included wildtype C57/BL6 mice as well as BAC transgenic mice expressing EGFP under the Mash1 genomic regulatory sequences (*Mash1^BAC-EGFP^*, referred to as Mash1-EGFP [22]). In this reporter mouse line, EGFP is authentically observed in Mash1-expressing cells and persists after initial expression of Mash1 [11], [23]. These mice were bred over wildtype C57/BL6 background for several generations and positive animals were selected at birth under UV light. For identification of stem cells by exclusion of fast cycling progenitors, EdU was given to the animals for 72 hrs in drinking water with 0.5% sucrose (1 mg/ml). Mice were sacrificed by injection with an intraperitoneal overdose of pentobarbital (Eutha77 in Ringer’s solution), followed by transcardial perfusion of 50 ml Ringer’s solution and 100 ml 4% paraformaldehyde (PFA) dissolved in phosphate buffered saline (PBS; pH 7.4). Following removal, brains were postfixed in PFA overnight at 4°C and cryo-protected by immersion in 30% sucrose dissolved in PBS. Brains were cut in coronal sections at 50 µm thickness, and serial sections collected from the olfactory bulb to the caudal regions of the LV (defined by the appearance of the dendate gyrus).

**Figure 4:**
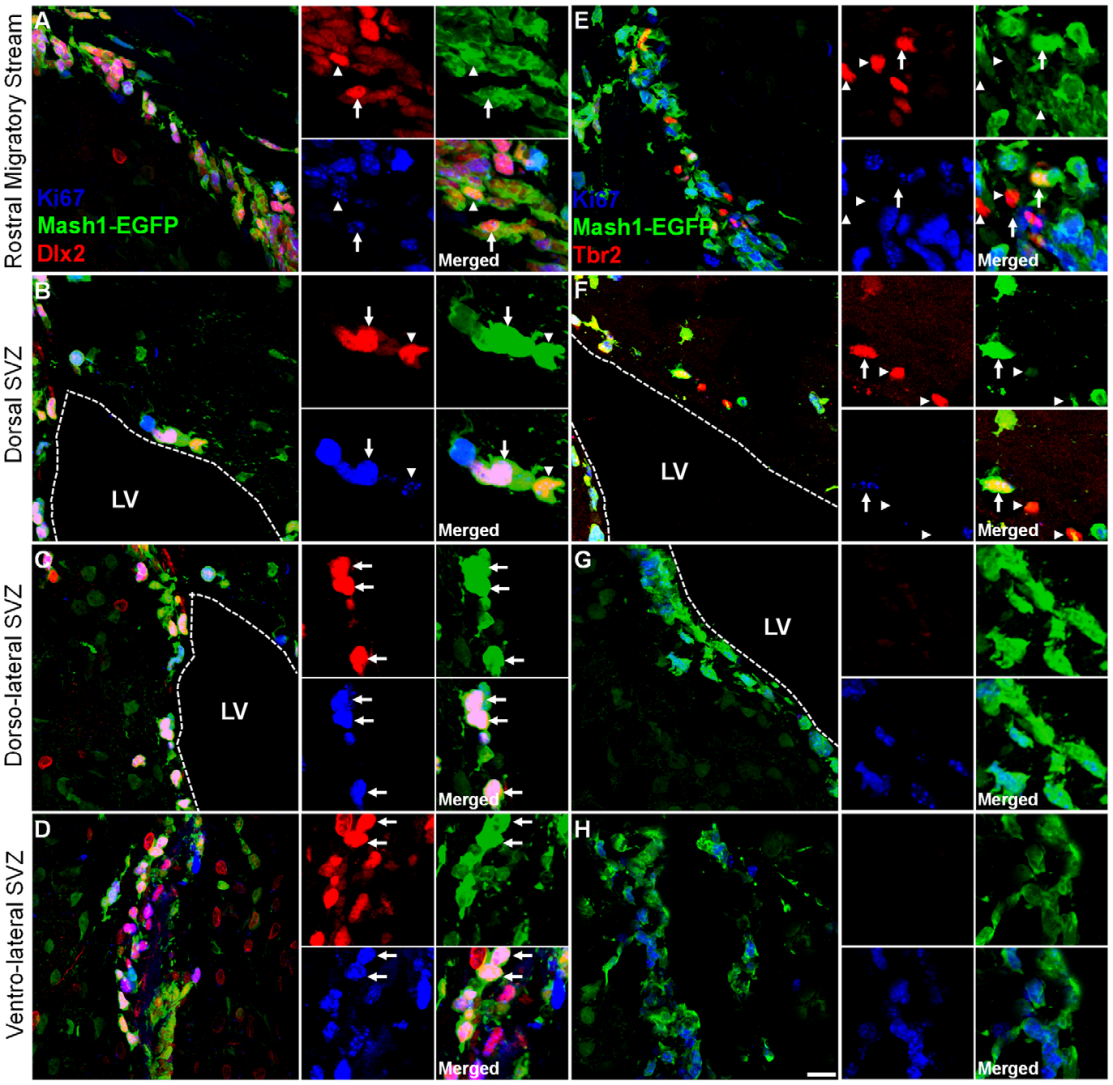
Dlx2 and Tbr2-progenitors originate from Mash1-positive type-C cells. Adult Mash1-EGFP+ mouse forebrain coronal sections at rostro-caudal point 1.2 relative to the bregma were immunostained for Dlx2 or Tbr2 (red), Ki67 (blue). Full panels are shown along with their single channel captions for examples. Top panels illustrate the RMS (**A,E**), whilst the dorsal SVZ (**B,F**), dorso-lateral SVZ (**C,G**) and ventro-lateral SVZ (**D,H**) are also shown. In the left side panels, Dlx2+ progenitors are readily visible by immunofluoresence in all regions examined, although more notably within the RMS (**A**) dorso-lateral SVZ (**C**) and ventro-lateral SVZ (**D**). Cropped captions of the different channels show examples of cells colocalising Dlx2+/EGFP+/Ki67+ (arrows) or Dlx2+/EGFP+ (arrow heads). In the right side panels, Tbr2+ progenitors are fewer and can be found migrating in the RMS (**E**) and also at the dorsal SVZ (**F**), but not in other SVZ microdomains (**G,H**). In some cases, arrowheads also show weaker levels of Ki67 staining in EGFP+ cells. In both the RMS (**E**) and dorsal SVZ (**F**), progenitors colocalising Tbr2+ with EGFP are frequently observed. While some Tbr2+ cells show high EGFP expression (arrow), low EGFP expression levels were observed in most Tbr2+ cells (arrow-head). Expression of Ki67 was observed in some Tbr2+ cells (**F**, arrow). Dotted lines mark the approximate ventricular space. Flattened confocal z-stacks are of 14–15 µm thickness, including captions. Scale bars: 15 µm in full panels and 20 µm in captions.

### Immunolabelling Procedures

Fluorescent labeling of cells in S-phase by EdU (5-ethynyl-2′-deoxyuridine) detection was performed following manufacturers guidelines using Click-it EdU Alexa Fluor 555 imaging kit (Invitrogen). Fluorescence and DAB immunostainings were performed as previously described [16]. Briefly, sections were incubated with primary antibodies at 4°C overnight (36 hrs for anti-GFAP). The following primary antibodies were used at the indicated dilutions: rabbit anti-Tbr2, 1∶1500 (Abcam, AB23345); guinea pig anti-Dlx2, 1∶5000 (Kuwajima et al 2006); mouse anti-Ki67, 1∶500 (BD Pharm, 550609); goat anti-Dcx, 1∶500 (Santa Cruz, Sc-8066); chicken anti-GFP, 1∶1000 (Abcam 13970); rabbit anti-ID1, 1∶500 (Biocheck, Bch-1 37-2); goat anti-Mcm2, 1∶300 (Santa Cruz, sc-9839). Sections were washed and incubated with species-matched alexa-488, -555 or 633-conjugated secondary (Invitrogen, Basel, CH) or biotinylated antibodies (Jackson Immunoresearch). For the latter, incubation with alexa-conjugated Streptavidin (EGFP or ID1 staining) was performed for 30 mins. Sections were counterstained with DAPI and/or Topro3 (Invitrogen) and cover-slipped with antifading mounting medium (Vectashield, Vector Labs).

**Figure 5:**
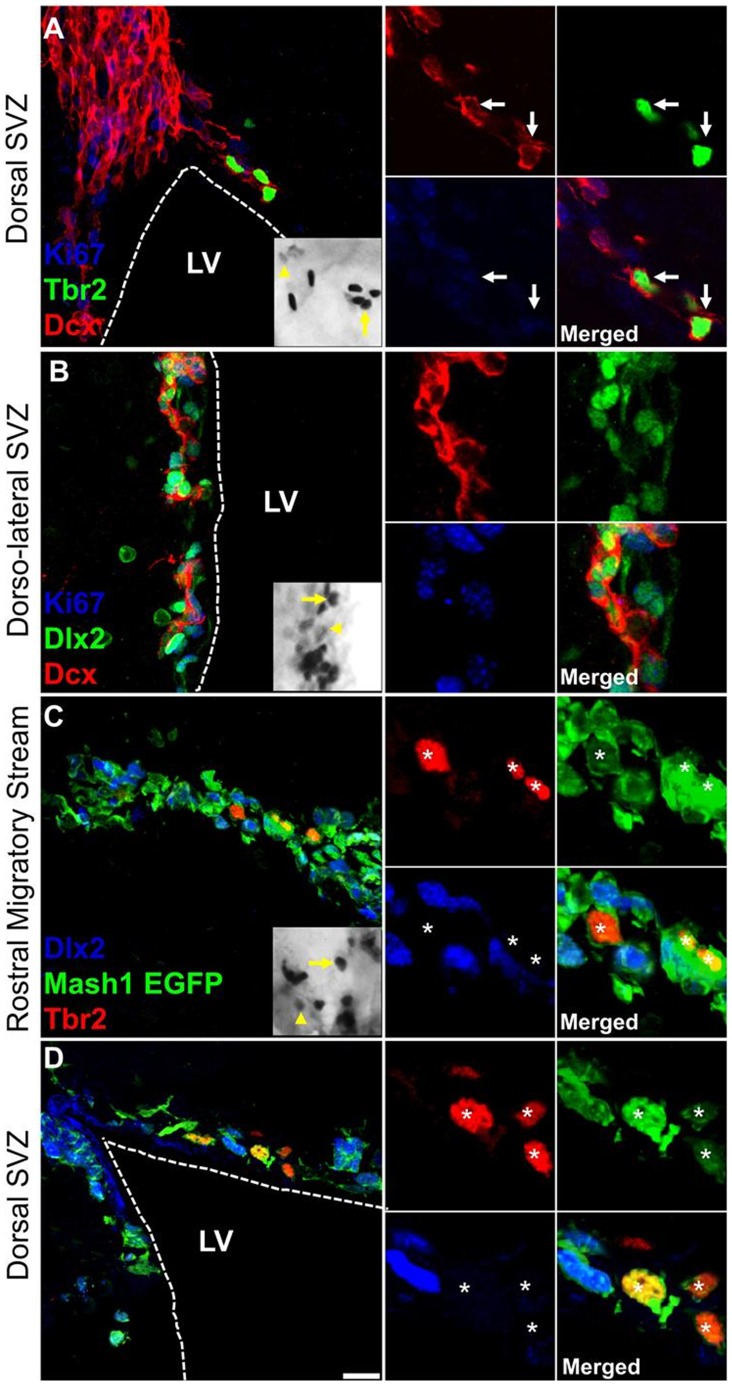
Dlx2 and Tbr2 identify non overlapping progenitor lineages in the adult mouse SVZ. (**A–B**) Adult C57/BL6 mouse forebrain coronal sections at rostro-caudal point 1.2 relative to the bregma were immunostained for Dcx (red), Dlx2 or Tbr2 (green) and Ki67 (blue). Both progenitor populations show characteristics of migrating neuroblasts, as indicated by their Dcx expression (**C–D**). Adult Mash1 mouse forebrain coronal sections at rostro-caudal point 1.2 relative to the bregma were immunostained for Tbr2 (red) and Dlx2 (blue). Both Tbr2 and Dlx2 exhibited EGFP expression, but showed no colocalisation. Right side captions show cropped individual channels and the merges. Full panel insets are zoomed and cropped DAB stained photomicrographs of rostral periventricular sections for Tbr2 in the dorsal SVZ (**A**), Dlx2 in the dorso-lateral SVZ (**B**) and Mash1 in the ventro-lateral SVZ (**C**). Yellow arrows and arrowheads show respectively positive stained cell and low level TF staining. Dotted lines mark approximate boundaries of ventricular space. Flattened confocal z-stacks are of 14–15 µm thickness, including captions. Scale bars: 15 µm in full panels, 20 µm in captions and 25 µm in insets.

For DAB immunostaining, sections were incubated in ABC solution at RT for 90 min in TBS according to manufacturer’s instructions (Vector Laboratories). Sections were incubated for 10 min in NiSO4 and DAB (20 mg/mL). The DAB reaction was activated by incubating sections in the latter solution containing 0.005% H_2_O_2_, monitored under a dissecting microscope, and reaction discontinued by washing sections thoroughly in PB when staining stopped intensifying. Dehydration was done by sequential immersion of slides containing stained sections in 70%, 80%, 100% EtOH and Xylol (20 s each) followed by a final wash in Xylol (2 min). Slides were immediately covered with Eukitt (Grale Scientific) and cover-slipped.

**Figure 6:**
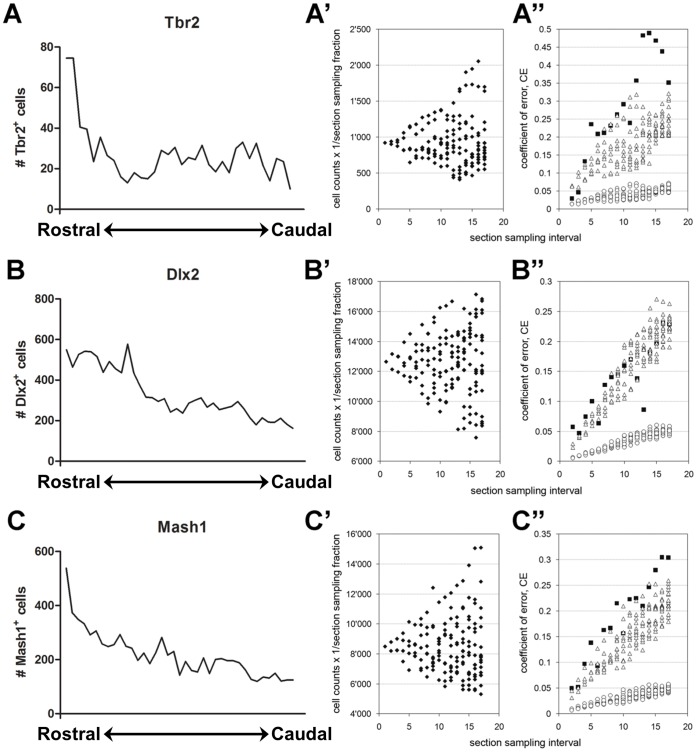
CE estimation of cells expressing Tbr2, Dlx2 and Mash1 in the adult mouse forebrain. (**A–C**) Left hand panel graphs showing the distribution of cells immunoreactive for the 3 markers in individual sections along the anterior-posterior axis of the forebrain. Only sections containing the LV were sampled. Rostral  = 1.2 relative to the bregma; Caudal  =  −1.2 relative to the bregma. (**A’–C’**) cell counts obtained for all 3 markers, by sub-sampling the original counts for section sampling intervals using every 2nd to every 17th sections. (**A”–C”**) estimates of the CE obtained by using Gundersen-Jensen estimator using a smoothness constant of 0 (open triangles) and 1 (open circles) for section sampling intervals using every 2nd to every 17th sections. Black squares indicate the observed CEs, i.e. the coefficient of variation among the replicates available for each sampling interval.

### Quantitative Procedures

The distribution of Ki67+, Mash1+, Dlx2+ and Tbr2+ cells was analysed on DAB stained sections. To study the rostro-caudal distribution of cycling cells (i.e. positive for Ki67), all cells in all sections, ranging from the olfactory bulb to the caudal regions of the LV (marked by the appearance of the dentate gyrus) were counted. The olfactory bulb and LV could be identified in a total of 68 sections. For analysis of the rostro-caudal distribution of cells positive for Mash1, Dlx2 and Tbr2, cells were counted in all sections encompassing the LV, i.e. on average 48 sections.

**Figure 7:**
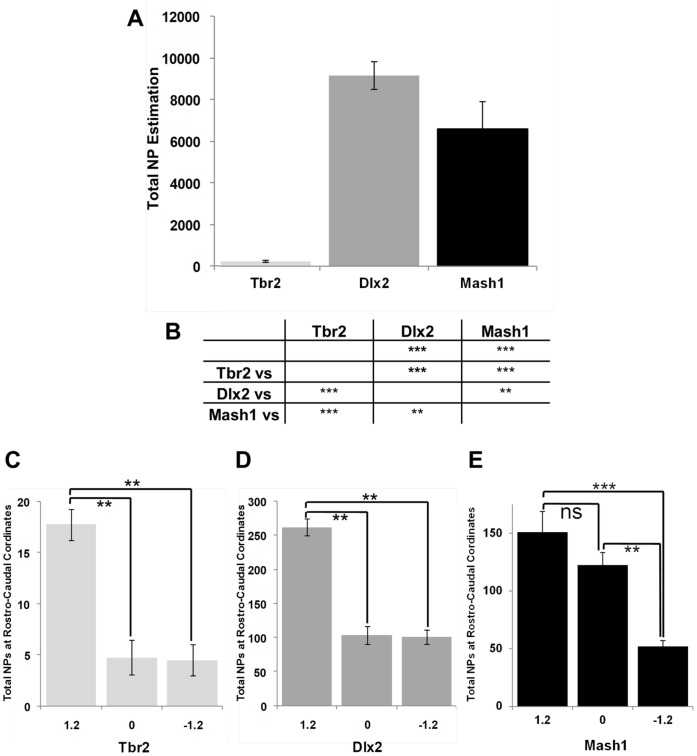
Population size and rostro-caudal distribution of cells expressing Tbr2, Dlx2 and Mash1 in the adult mouse forebrain. (**A**) For each marker, numbers from each of the SVZ microdomains summed and multiplied by the inverse of the section sampling fractions, i.e. 12 for Mash1 and Dlx2 and 6 for Tbr2. The average (n = 5) and their SD were plotted. (**B**) Table summarizing statistical comparisons of estimates of each maker. (**C–E**) For each animal, the combined numbers of cells quantified in the SVZ at rostro-caudal axis points 1.2, 0 and −1.2 relative to the bregma were compared within these rostro-caudal axis points. ns: no significance; *p<0.05; **p<0.01; ***p<0.001.

To determine the influence of section sampling interval on the precision of estimates of cell numbers, samples of sections were drawn at incrementally higher sampling intervals from the exhaustive series of sections [24]: up to every 17^th^ section for Ki67, Mash1, Dlx2 and Tbr2. For every sampling interval from 1 to 17, cell number estimates were calculated for all possible samples, e.g., five estimates were calculated for samples using every 5^th^ section. The coefficient of variation (standard deviation of the estimates obtained from all subsamples of a sampling interval divided by mean of the estimates) was calculated as an empirical estimate of the precision to be expected from a particular sampling interval.

**Figure 8:**
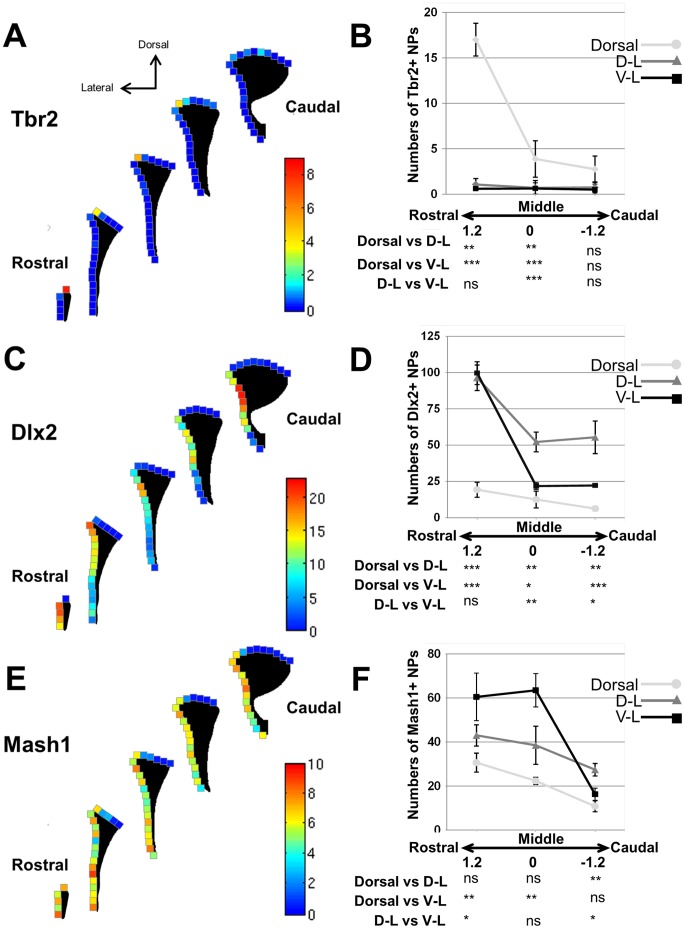
Progenitors expressing Mash1, Dlx2 and Tbr2 define distinct dorso-ventral microdomains of the adult mouse SVZ. (**A,C,E**) Heat maps showing the densities (from higher (red) to lower (blue) values) of quantified progenitors in 100 µm sized squares positioned along the dorsal and lateral LV walls at 5 representative rostro-caudal coordinates (1.2 as rostral, 0.6, 0, −0.6 and −1.2 as caudal). Counts and statistical analysis of the dorso-ventral distribution of Tbr2+ cells (**B**)**,** Dlx2+ cells (**D**) and Mash1+ cells (**F**) at defined rostro-caudal coordinates of the LV (rostral = 1.2; middle = 0; caudal = −1.2). Statistical analysis (Bonferroni’s followed by posthoc test) below histograms at rostro-caudal axis 1.2, 0 and −1.2 significance of quantified progenitors in individual SVZ microdomains. D-L  =  dorso-lateral; V-L  =  ventro-lateral ns  =  no significance; *p<0.05; **p<0.01; ***p<0.001.

Empirical estimates were compared to CE (coefficient of error) estimates calculated using the Gundersen-Jensen (G-J) CE estimator [25], which aims at providing an estimate of sampling precision based on only one available sample. The G-J CE estimator has been shown to provide useful estimates of the CEs of cell number estimates in complexly shaped structures with spatially unevenly distributed cell populations [24]. The number of cells contained in each section represented an exhaustive count. Consequently, variance originating from the sampling within section, *S* ^2^, was omitted from the G-J CE estimator. A smoothness factor, *m*, is part of the CE estimator. This factor accounts for the different estimate variances to be expected from smooth (*m* = 1) or jagged (*m* = 0) distributions of counts derived from individual sections along the axis of sectioning. Although an *m* of 1 has been suggested preferable [25], this may not always be the case [24]. Consequently, G-J CE estimates were calculated for a smoothness factor, *m*, of 0 and 1. For the subsequent quantification of cell populations, cells expressing high levels of Ki67, Mash1, Dlx2 and Tbr2 were counted in uniform systematic samples of sections. Based on the empirical data on estimate precision (see Results) we selected a sampling interval of 12 for Ki67, Mash1, Dlx2 and 6 for Tbr2 to ensure that CEs of the estimates would likely be below 0.2. Cells in the top focal plane of the section were excluded from the counts. All positive cells in the dorsal and lateral walls of the LV were counted. Dorsal SVZ was considered when adjacent to the corpus callosum. Cells in the dorso-lateral corner of the LV were excluded from any quantification as they represent part of the RMS. Positive cells within 100 µm distance of the ventricle boundary were quantified.

The distribution of slow cycling NSCs was analysed on fluorescent stained sections following a method described elsewhere [18]. Briefly, Mcm2 a protein of the pre-replication complex that identify both cycling and non-cycling cells with proliferative potential was used together with the S-phase marker EdU. Slow cycling NSCs (Mcm2+/EdU− cells) were quantified by process of elimination of EdU incorporation over a 3 day period. Mosaics of individual sections were acquired using an Olympus FluoView FV1000 confocal microscope equipped with a motorized stage. A 20x UPLSAPO objective was used (N.A. 0.75) to capture mosaic stacks encompassing the entire dorsal and lateral walls of the LV. Individual stacks were acquired at 800/800 px resolution with a step size of 1.5 microns, and merged using the integrated module of the FluoView software. The obtained mosaics were transformed to RGB Tiff series using the Imaris software (Version 7.3; Bitplane) and imported to Neurolucida where the LV contours and position of individual Mcm2+/EdU− cells were drawn.

### Heat Maps Design

For cycling cells (Ki67+) and slow (or infrequently) cycling NSCs (Mcm2+/EdU−), 100 µm side square contours were superimposed to the neurolucida drawing for each individual section from the appearance of the LV (1.2) to the appearance of the dentate gyrus (−1.2). Squares were placed along the dorsal and lateral aspects of the LV starting from the dorso-lateral corner. The number of cells contained in each individual square was extracted by using the “number of marker in closed contour” function of the neurolucida explorer software, for each section. To generate a heat map, conditional formatting of cells was applied in MATLAB (R2011b, Mathworks, CH). Cells were formatted based on their values using a multi-color scale (blue lower value, red higher value). Heat maps for Mash1+, Dlx2+ and Tbr2+ progenitors were only generated for 5 equally spaced sections at selected rostro-caudal coordinates (1.2 to −1.2 relative to bregma). A minimum of 3 animals were quantified. Briefly, 100 µm side squares were superimposed to neurolucida drawing as described above. Cell counts obtained for each animal were averaged, and a heat map was generated as described above.

### Transcription Factors Expression Pattern Analysis

Co-expression of selected transcription factors (Mash1, Dlx2, Tbr2, Id1) with proliferative (Ki67, Mcm2, Edu), neuroblasts (Dcx) or NSCs markers (Id1, GFAP) was examined by double or triple immunofluorescent stainings on 5 equidistant sections spanning the entire LV. Co-localisation was assessed by confocal 3D reconstruction on a Leica SPE II or an Olympus FluoView FV1000 confocal microscope equipped with a 40x objective (N.A. 1.25). A minimum of 100 cells in at least 3 individual animals were analyzed.

Expression of Mash1-driven EGFP in Tbr2+ or Dlx2+ progenitors was quantified by examining rostral periventricular sections (where most Tbr2+ progenitors are located) for immunolabelling with Tbr2 or Dlx2 as well as the nuclear counterstain Topro3. For each animal, more than 20 Tbr2+ cells in the dorsal SVZ or Dlx2+ cells in the dorso-lateral SVZ were randomly selected maintaining equal acquisition parameters for obtaining mean volume pixel intensities of EGFP within Tbr2+/Dlx2+ progenitors. For Dlx2+ progenitors, the left hemisphere was followed as above whereas for Tbr2+ progenitors, both hemispheres were analysed to yield constant cell numbers examined between animals. EGFP densitometry was performed using the Leica software supplied following the manufacturers guidelines.

Unless stated, all quantifications are presented as mean and the standard error of the mean (± SEM), and samples were compared for significance using ANOVA followed by Bonferroni’s post-hoc test or unpaired t-test (Prism v3.02 software, GraphPad).

## Results

### Distribution of Cycling Cells in the Adult Forebrain Germinal Zones

We first performed a 3D analysis of the distribution of proliferating cells of the adult forebrain. The entire population of cells showing Ki67-immunoreactivity was marked and superimposed onto schematic drawing delineating the forebrains and LV outlines. This was applied for all coronal sections collected between the olfactory bulb and caudal regions of the LV, as illustrated by the volumetric modelling shown in **Fig. 1A–B**. Numerous proliferating, Ki67+ cells could be observed at all rostro-caudal coordinates, including the RMS, although with marked quantitative differences. The rostro-caudal distribution of Ki67+ cells revealed a sharp peak rostral to the LV, where migratory neuroblasts originating from the LV walls merge to migrate through the RMS (**Fig. 1B**). In the LV, the numbers of Ki67+ cells appeared greater within the most rostral regions of the LV relative to caudal regions. Heat maps for Ki67+ progenitors further illustrated the rostral enrichment and homogeneous distribution of proliferating cells throughout the lateral wall of the LV (**Fig. 1C,D**). This contrasted with the dorsal wall where proliferating cells were evident in its lateral most regions, whilst only few Ki67+ cells could be observed in more medial regions (**Fig. 1C**).

To analyse the consequences of this uneven distribution on cell number estimates obtained from statistically representative samples of sections, we next estimated total cell numbers by using section sampling intervals ranging from every 2^nd^ to every 17^th^ section (**Fig. 1D** **’**). The corresponding observed CEs, based on the empirical data, were compared to CE estimates based on the Gundersen–Jensen estimator using smoothness factors of 0 and 1 (**Fig. 1D** **”**). A smoothness factor of 0 provided a good estimation of the observed CEs. In contrast, a smoothness factor of 1 underestimated the observed CEs by a factor of ∼4. CEs rise steeply with increasing sampling intervals. To obtain, e.g., a CE of ∼0.05 would require the exhaustive counting of cells in every 5^th^ section.

### Detection and Distribution of Slow Cycling Cells in the SVZ

While proliferation markers such as Ki67 label type-B and mainly type-C cells that are both sessile [26], they also label a small population of migrating neuroblasts (∼15%; [4]). Due to this non-specificity, we next focused our attention to specific markers of SVZ-NSCs and of progenitors of independent lineages (Tbr2 and Dlx2; see below). For detection of slowly cycling NSCs, Mcm2 a protein of the pre-replicative complex was used in combination with a S-phase marker (i.e. EdU), as described previously [18] (**Fig. 2A–D**). Compared to label-retaining protocols, applying Mcm2 with EdU has the advantage to exclude from the quantification, cells that would artifactually withdraw from the cell cycle due to potential analogue toxicity [18]. Comparison of the pattern of Mcm2 and Ki67 confirmed co-expression of the 2 markers in >97% of cycling cells (440 cells counted in 3 animals, **Fig. 2B, E**). Administration of the S-phase marker EdU to the animals for 3 days (through drinking water) resulted in the labelling of a larger population of actively proliferating cells compared to Ki67-positive cells alone (i.e. type-C and to a lesser extent type-A cells; **Fig. 2A,B**). About half of these cells (i.e. 47.4±4.2%, 859 cells quantified in 3 animals) were negative for Mcm2, indicating that they had exited cell cycle during the 3-days treatment period. Among Mcm2+ cells, 93.8±0.6% (440 cells quantified in 3 animals) had incorporated EdU, while 6.2% ±0.6%) were negative for this S-phase marker indicating that they had not cycled during the treatment period, and can therefore be defined as slow or infrequently cycling cells (**Fig. 2E**). We next focused our analysis onto these Mcm2+/EdU- cells which were located directly adjacent to the ependymal wall. Their potential NSC identity (i.e. type-B cells) was confirmed by co-expression of proposed stem cell markers markers, i.e. Id1 and GFAP [27], [28] (see **Fig. 2A, C**). Quantification with Id1 showed that ∼9% of Mcm2+ cells were Id1+, **Fig. 2C**, a majority of which were EdU− or Ki67- (**Fig. 2B**). Immunodetection of Mcm2 and EdU in Mash1-EGFP animals confirmed that about 60% of the Mcm2+/EdU− expressed no EGFP, while the other 40% were weakly positive, reflecting the low level of Mash1 expression previously described in type-B cells [29]. These results show that Mcm2+/EdU− cells indeed present the cyclic behaviour and antigenic properties of conventionally defined type-B cells, and are thus referred to as NSCs throughout this study.

Throughout the rostro-caudal axis of the LV, greater densities of NSCs were detected along the lateral aspect of the dorsal wall and homogeneously throughout the lateral SVZ (**Fig. 2G**). The rostral enrichment of NSCs was apparent in all of the subregions of the SVZ as presented in heat maps (**Fig. 2G**) and in the rostral photomicrographs shown in **Fig. 3A–C**. Due to their low density, heat maps for NSCs, showed a patchy distribution, although their general distribution was comparable to those of Ki67+ cells (compare **Fig. 1C** **with** **Fig. 2G**). This comparable distribution was further confirmed by quantifying the proportion of Mcm2+/Edu− cells among the entire population of Mcm2+ cells at 3 rostro-caudal levels (i.e. 1.2, 0 and −1.2 to bregma) in 4 animals. At these 3 levels, the proportion of NSCs among all cycling cells remained constant (p>0.2; **Fig. 2F**).

### Dlx2 and Tbr2 Label Distinct Populations of Mash1-derived Progenitors in the Adult SVZ

We next estimated the degree of co-expression of Ki67 with Dlx2+ or Tbr2+ progenitors in the *Mash1^BAC-EGFP^* mouse, where the EGFP persists after initial Mash1 expression allowing visualization of their early progeny. The specificity and reliability of this reporter line has been confirmed previously [11]. The RMS and the different SVZ compartments of rostro-caudal periventricular sections were analysed, but the most rostral periventricular sections were followed in greater detail as Dlx2+ and Tbr2+ progenitors are located in greater densities in these regions (note: intense DAB staining of all progenitors in the most rostral regions compared to caudal regions of **Fig. 3G–I** **and** **Fig. 4**). In the RMS and along the entire rostro-caudal axis of the LV, numerous Dlx2+ progenitors were observed. As described previously, a significant proportion of these progenitors were positive for Ki67 [14], **Fig. 4A–D**). Unlike Dlx2+ progenitors, Tbr2+ progenitors were fewer throughout the rostro-caudal extent of the LV. Again, Tbr2+ cells were frequently observed to express Ki67 (arrowheads, **Fig. 4E, F**), in agreement with our previous report [11]. A significant population of both Dlx2+ and Tbr2+ progenitors also expressed the committed neuronal marker Dcx (**Fig. 5A,B**). Both Dlx2+ and Tbr2+ progenitors were EGFP+ in the *Mash1^BAC-EGFP^* mouse (Dlx2: **Fig. 4A–D** **,** see also **Fig. 5B**; Tbr2: arrows **Fig. 4E,F** **,** see also **Fig. 5A**), indicating that they express, or have expressed shortly before, the TF Mash1.

Finally, potential overlaps of Dlx2+ and Tbr2+ progenitors in the adult SVZ was studied in the Mash1-EGFP mouse line. No colocalization was observed, neither in the RMS nor in the dorsal SVZ, where a potential overlap of these progenitor pools would exist (**Fig. 5C,D**). Taken together, these observations demonstrate that Dlx2 and Tbr2 expression define divergent pools of progenitors that both derive from type-C cells showing different expression levels of Mash1. These two TFs can therefore be used to identify distinct early lineages in the adult SVZ.

### Distribution and Population Estimates of Cells Expressing Mash1, Dlx2 or Tbr2 in the Adult Mouse SVZ

We next assessed the distribution of cells expressing Tbr2, Dlx2 and Mash1 that identify divergent neurogenic lineages during development [30] as well as in the adult mouse forebrain (see above) [11]. There markers were focused on further in LV periventricular sections LV by DAB immunostaining (**Fig. 3G–I**).

As observed for Ki67 counts (see above), all three markers showed a clear gradient, with greater numbers of positive cells in the rostral-most compared to more caudal regions (**Fig. 3G–I** **and** **Fig. 6A–C**). We next estimated cell numbers obtained with sampling intervals ranging from every 2^nd^ to every 17^th^ section (**Fig. 6A** **’, B’ & C’**). The corresponding observed CEs, based on the empirical data, were compared to CE estimates based on the Gundersen–Jensen estimator using smoothness factors of 0 and 1 (**Fig. 6A** **”, B” & C”**). A smoothness factor of 0 again provided a good estimation of the observed CEs for all markers. In contrast, a smoothness factor of 1 underestimated all observed CEs by a factor of more than 5. Due to the smaller size and uneven distribution of Tbr2+ progenitors along the rostro-caudal axis, CE values increased more notably for this marker than for Mash1 or Dlx2 (**Fig. 6A** **”, B” & C”**).

All progenitor populations showed clear uneven distributions along the rostro-caudal axis in the LV. These uneven distributions impose constraints on the design of sampling schemes that can estimate the size of defined progenitor populations in the adult SVZ with a pre-determined level of precision. For the purpose of this quantitative study, we judged that a CE of less than 0.2 would be satisfactory. Empirical CE estimates indicated that every 12^th^ section should be analysed to obtain this precision for Ki67, Mash1 and Dlx2 cell numbers, while every 6^th^ section needed to be sampled for Tbr2+ progenitors. The RMS of periventricular sections (upper left corner of the SVZ) was excluded from these counts. In all cases and subsequent analysis to follow, only the left hemisphere was used for analysis. However, comparison of the left and right hemisphere of the individual SVZ microdomains through the rostro-caudal axis revealed that there were no significant differences between the hemispheres. Relatively few (Ki67+/Mash1+/Dlx2+) cells were found in medial SVZ regions only, which were not included in these quantifications. Estimates of the average numbers (standard deviation in parentheses) of progenitors per animal were 236 (52.7) for Tbr2, 9156 (665) for Dlx2 and 6600 (1302) for Mash1 (**Fig. 7A, B**). Estimates for the total numbers of Ki67+ progenitors were 6105 (451) which was not significantly different (p>0.8) from Mash1+ progenitor total numbers but different from Tbr2 and Dlx2+ progenitor cell numbers (p<0.001; ANOVA followed by Bonferroni’s posthoc test). This coincides with the observation that Ki67 and Mash1 expression is present in common progenitors as illustrated by the strong overlap of Ki67 with EGFP in the *Mash1^BAC-EGFP^* mouse (**Fig. 4**). There were on average 39 times more Dlx2 than Tbr2 progenitors (p<0.001) **(** **Fig. 7A,B**), illustrating the very pronounced differences in the size of these two independent lineages.

In order to further investigate the rostro-caudal distribution of cells expressing these markers, quantification was performed at rostro-caudal axis points 1.2, 0 and −1.2 relative to the bregma for comparison (**Fig. 7C–E**). These analysis revealed that there were significant differences (p<0.01 for all markers) in the numbers of cells located at the levels specified, with the highest numbers found in rostral regions of the LV (**Fig. 7C–E**). As a whole, based on these rostro-caudal axis points, greater than approximately 60% of all progenitors studied are generated rostrally.

### Progenitors Expressing Tbr2, Dlx2 and Mash1 are Enriched in Defined Walls of the LV

We next assessed by DAB immunolabelling the distribution of Tbr2, Dlx2 and Mash1-expressing cells in 3 dorso-ventral regions of the LV: i.e. defined as dorsal, dorso-lateral and dorso-ventral microdomains. In order to concentrate our analysis on progenitors expressing these markers and discard their immediate progeny, we analysed cells with moderate to high TF expression (compare arrows versus arrowheads in insets **Fig. 5A–C** **)**. Again, the RMS of periventricular sections (upper left corner of the SVZ) was excluded from these counts as this region contains large numbers of migrating DCX+ neuroblasts (**Fig. 5B**). Rostro-caudal representative examples of DAB immunolabelling of markers in different subregions of the SVZ are presented in **Fig. 3G–I**. DAB immunolabelled sections show that all 3 TFs studied were eachconfined to subregions (or microdomains) of the LV, and showed relative anterior enrichment as illustrated by heat maps (**Fig. 8A, C, E**). Further quantification showed that Tbr2+ progenitors were clearly confined to the lateral region of the dorsal SVZ (**Fig. 8A, B**). Dlx2+ progenitors appeared highly enriched in the dorso-lateral SVZ (**Fig. 8C, D**) while Mash1+ progenitors were more uniformly distributed along the lateral SVZ subregions **(** **Fig. 8E, F** **)**. For this later population, a relative enrichment in ventral most regions was however noticed, which was due to higher Mash1 expression levels in ventral progenitors. This was confirmed by densitometric analysis where the measured expression intensities of EGFP was approximately 2-fold lower in Tbr2+ (±5.3% SEM; 68 cells; n = 3) than Dlx2+ progenitors (±11% SEM; 79 cells; n = 3) to highlight different levels of Mash1 expression by these two populations of progenitors.

## Discussion

Recent evidence indicate that adult NSCs located in defined SVZ regions of the postnatal and adult LV are biased to acquire specific fates [8]–[10], [31]. This regional diversity in NSC fate is thought to be coded by intrinsic properties, such as the expression of lineage directing TFs in their progeny [8], [10], [12]–[14]. In this study, we analysed the distribution of cell populations showing distinct cycling behaviours (i.e. rapid vs. slow cycling cells) and progenitor populations expressing lineage-specific TFs were unevenly distributed spatially in microdomains that surround the LV. This emerging SVZ complexity prompted us to develop optimal sampling schemes that will facilitate efficient estimates of defined NSCs/progenitors populations’ distribution and sizes.

### Location and Distribution of Cycling Cell Populations in the SVZ

As NSCs (type-B cells) are relatively quiescent and are directly located at the walls of the LV, they are surrounded by fast proliferating cells (type-C cells) and migrating neuroblasts (type-A cells) [1]. With the exception of cycling cells (see below), little information exists for the size of distinct populations of SVZ-NSCs/progenitors in the adult SVZ. Here, we present for the first time design-based stereological estimates of NSC numbers and their subsequent progenitor population sizes, where previously crude numbers have been compiled at most. Our work reveals that ∼12,200 cells are cycling (i.e. express Ki67+) at a given time in the LV of young adult (2.5 month old) mice (i.e. ∼5100 cells per hemisphere). This number is in the range reported in other studies [32]–[34]. Our data’s reveal a clear rostral enrichment of cycling cells compared to more caudal regions of the LV. This distribution might be partly due to the rostral migration of neuroblasts, a fraction of which proliferate (∼15%; [4]). In comparison to cycling cells, detailed analysis of SVZ-NSC numbers and of their dissemination through the rostro-caudal axis of the LV is scarce. This has been due to difficulties in detecting NSCs with the use of a single marker [29], [35]. Previous studies however proposed that NSCs can be distinguished from surrounding proliferating cells because of different cell cycle kinetics [18], [36], [37]. Immunodetection of Mcm2, a protein of the pre-replicative complex has been used to identify non-cycling cells with proliferative potential in a variety of contexts [38]. Combination of this marker with nucleotides analogues (e.g. EdU) that label cells in S-phase, allow the identification of slow or infrequently proliferating cells (i.e. Mcm2+/EdU-) by way of fast proliferating cell elimination (i.e. Mcm2+/EdU+) [18]. Here, we’ve used this previously established protocol, instead of long-retaining label protocols that present their own caveats [18], [39]. This allowed us to label and exclude cells proliferating over a 3 day period to identify and quantify NSCs (slow or infrequently proliferating cells) throughout the rostro-caudal extent of the adult SVZ. Our results, showing frequent immunodetection of GFAP and Id1, two previously proposed adult NSC markers [27], combined with the low or absent expression of Mash1 in Mcm2+/EdU- populations, support the NSC nature of these cells [29]. In applying these strict labelling criteria, our results show a similar rostro-caudal distribution of Mcm2+/EdU- NSCs in comparison to cycling Ki67+ cells. This similar distribution was further supported by our quantifications showing robust co-expression of Mcm2 and Ki67 in most cells and stable ratios of Mcm2+/EdU- vs. Mcm2+/EdU+ cells observed at 3 representative rostro-caudal levels of the LV. Thus, our quantifications consistently show that ∼6% of all cycling cells are slow or infrequently cycling (i.e. Mcm2+/EdU-). Extrapolation of these numbers from our quantification of Ki67+ cells suggest that the adult SVZ contains ∼600 to 750 NSCs, a number in line with previous estimates obtained from larger sampling regions [40], [41]. Furthermore, our observations of a rostral enrichment of cycling cell populations relative to more caudal LV regions are in agreement with the greatest neurosphere forming capacity of SVZ-NSCs occurring in rostral sections and declining markedly in caudal regions or from the middle of the LV [39].

### Expression of Unique TFs Define SVZ Microdomains

During development, most olfactory bulb interneurons originate from progenitors located in ventral regions of the telencephalon, namely from the LGE [42], and depend on the expression of Mash1 and Dlx2. In contrast, pallial regions contain progenitors that express Tbr2 and contribute to glutamatergic neurogenesis in the cortex [43] and olfactory bulb [44]. We next explored further this uneven distribution in the adult SVZ. Quantification of progenitors expressing selected TFs, i.e. Mash1, Dlx2 and Tbr2 led to interesting observations. First, all markers showed a clear rostral enrichment coinciding with cycling cell locations. Largely Mash1+ progenitors as well as proportions of Dlx2+ and Tbr2+ cells contained characteristics of progenitors cells (i.e. Ki67+/Dcx−), a population of cells known to be sessile [26]. Dramatic differences existed in the numbers of lineage specific progenitors. Our estimations reveal that Dlx2+ progenitors are generated approximately 39-fold more often than Tbr2+ progenitors and Dlx2+ progenitors are expanded further by 1.5-fold compared to Mash1+ progenitors from which both types arise [11], [45]. Based on the expression of these 3 TFs, our 3D maps reveal the existence of 3 dorso-ventral microdomains of the LV walls. A dorsal domain that is enriched in progenitors expressing the TF Tbr2, as well as a dorso-lateral domain that are enriched in progenitors expressing Dlx2, and a ventro-lateral domain enriched for Mash1 expressing progenitors. The observed bias in enrichment for Mash1-expressing progenitors appear to be due to higher expression level of this TF in ventral progenitors compared to dorsal progenitors as shown by our densitometric measurement in Tbr2 and Dlx2 progenitors. Nevertheless, the clear spatial segregation of progenitors positive for Tbr2 and Dlx2 (and Mash1 to a lesser extent) to distinct dorso-ventral microdomains of the LV, further support the emerging view that major aspects of embryonic patterning are preserved in the adult SVZ [3].

The rostral enrichment of NSCs/progenitors may rely on the flow of the cerebral spinal fluid that is produced caudally from chloroid plexuses [46]. It is likely that morphogens secreted by the choroid plexus influence also the distribution and cycling behaviour of NSCs/progenitors located in most rostral SVZ regions [47]. The mechanisms involved in the establishment and maintenance of distinct dorso-ventral SVZ microdomains remains elusive. In this context, it is interesting to note the detection of the morphogen Shh in ventral regions of the adult SVZ, which was shown to be responsible for the generation of deep granule neurons and calbindin-positive periglomerular cells of the olfactory bulb [48]. This role for morphogen programs in the establishment and maintenance of SVZ organisation remains to be fully explored.

### Implication for the Precision of Estimates of SVZ Progenitor Populations

The vast majority of CNS cell populations are **so large that cell numbers must be estimated from an extrapolation of cell counts made in** statistically representative samples of the populations. In addition to variance caused by true differences between individuals (biological variance), sampling inevitably introduces methodological variance. The sum of the biological and methodological variance is part of practically all common statistical procedures that aim at testing competing hypotheses in experimental research. Thus, if the methodological variance is too large, it might mask significant **differences** between experimental groups, resulting in false-negative results and conclusions. CE estimators represent a tool to evaluate such possibility by providing an estimate of the size of the methodological variance resulting from a particular sampling scheme.

Quantitatively, the SVZ is, due to its thinness, irregular shape and dramatic heterogeneous distribution of the cell types assessed in this study, an ‘ill-behaved’ structure. The precision of cell number estimates decreases rapidly with increases in section sampling intervals. The degree of precision in this study, a CE of individual estimates of ∼0.2, was acceptable for the following reasons. A CE of ∼0.1, a value aimed for in many descriptive studies, would require exhaustive counts in sections sampled at intervals of four or lower for Mash1 and Dlx2 and three or lower for Tbr2 (**Fig. 6A–C**) - a prohibitive workload for quantification of Mash1+ and Dlx2+ progenitors. Sampling a still very labor-intensive subset of, e.g., 1000 cells to limit workloads would add ∼0.03 to the CE. When aiming for combined CE’s of 0.1, this allows only 0.07 to originate from the sampling of sections, which would require section sampling intervals of three or less for Mash1 and Dlx2, i.e. the analysis of 16 or more sections in which 1000 cells are sampled for each marker. Using two-tailed t-statistics and 0.15 as the biological contribution to group variance, a ∼20% effect could be detected in an experiment using such a sampling scheme and 5 control and 5 experimental subjects. The sampling scheme used in this study required the quantification of far fewer cells in only one-quarter of the sections which would allow the detection of a ∼20% effect in groups containing 8 subjects each. This represents an increase in the workload of 60% in terms of the subjects used in comparison to the increase of more than 400% in terms of sections to be analysed and cells quantified when aiming for CE’s of 0.1 in groups of 5 subjects each. Thus, in the SVZ, an increase in the number of subjects per group is by far more efficient in increasing statistical power than increasing the precision of the estimates to CE values below 0.2.

Accurate NSC/progenitor identification will prove to be important in several instances, for example assessing changes in NSC/progenitor populations or diversity in other mammalian species as illustrated by our recent work in primates [16]. Furthermore, in the context of brain repair following a lesion or various pathologies, endogenous NSCs with specific phenotypes might be recruited to replace lost neurons. NSCs as well as progenitors react to an injury by increasing their proliferation and migration to the injury site in its models of brain trauma [49]–[51]. In this perspective, the diversity of progenitors that transpire from the walls of the LV raises important questions. As microdomains of the LV are populated by different pools of NSCs which are biased to acquire specific fates, the differential response of these progenitors expressing distinct TFs may be investigated. Studies that will allow assessing if appropriate populations of progenitors respond to specific injuries will be necessary to judge the regenerative or replacement potential of specific neuronal subtypes, a process that is yet controversial. Although it has been suggested that adult SVZ progenitor cells can differentiate into neurons with region-appropriate phenotypes within damaged areas [52], [53], more recent studies challenge this view by showing that brain injury does not alter the intrinsic differentiation potential of adult neuroblasts [54]. A quantitative assessment of divergent progenitor population will be important in further defining the potential of endogenous SVZ-NSCs in brain repair.
